# Understanding the Will Rogers Phenomenon in Cholangiocarcinoma Research and Beyond

**DOI:** 10.3390/cancers17193263

**Published:** 2025-10-08

**Authors:** Ruslan Akhmedullin, Zhandos Burkitbayev, Tair Koishibayev, Zhanat Spatayev, Abylaikhan Sharmenov, Oxana Shatkovskaya, Dinara Zharlyganova, Almira Manatova, Zhuldyz Kuanysh, Sanzhar Shalekenov, Abduzhappar Gaipov

**Affiliations:** 1 Department of Medicine, Nazarbayev University School of Medicine, Astana 010000, Kazakhstan; ruslan.akhmedullin@nu.edu.kz (R.A.); abduzhappar.gaipov@nu.edu.kz (A.G.); 2 National Research Oncology Centre, Astana 010000, Kazakhstana.sharmenov@erasmus.nl (A.S.);

**Keywords:** cholangiocarcinoma, Will Rogers phenomenon, surgical outcomes, survival, misclassification

## Abstract

**Simple Summary:**

The authors performed a comparative analysis of different cholangiocarcinoma (CC) subtypes and provided a reclassification analysis to investigate the impact of misclassifications of Klatskin tumors in CC research. Overall, our study revealed weak evidence of survival differences between the subtypes after curative liver resection. We also questioned whether the Will Rogers phenomenon is supported by statistical evidence when contrasting changes that come from multiple reclassifications in the cancer literature.

**Abstract:**

Background. The existing literature highlights a lack of comparative studies between subtypes of cholangiocarcinoma (CC) and the impact of misclassification on the epidemiological parameters. Methods. A retrospective study was conducted to evaluate the surgical outcomes. The authors used Poisson regression with modified errors to calculate the risk ratios (RR) and reported post-estimation marginal effects. Coefficient estimates, variance inflation factors, and Pearson’s goodness-of-fit test statistics were used to check for multicollinearity and model fit, respectively. We also performed a reclassification analysis by modeling Klatskin tumors (PCC) as extrahepatic (ECC), reclassifying them as intrahepatic (ICC), and comparing the corresponding changes in estimates. Results. Regression analysis revealed an increased risk of death in patients with ICC (RR = 2.05, 95% CI 1.11–3.78) and PCC (RR = 2.03, 95% CI 0.97–4.24) compared to those with DCC. When PCC was analyzed as an ECC, the ICC revealed an RR of 1.52 (95% CI 0.84–2.73). Further reclassification of PCC showed an RR of 2.04 for ICC (95% CI: 1.53–3.53). The adjusted marginal effects saw a reduction in the death probability for both ICC and ECC. However, post hoc analyses revealed insufficient evidence for differences between the reclassified models. Conclusions. Patients with DCC had slightly better prognosis compared to ICC and PCC. We found no differences in survival between ICC and ECC (combining DCC and PCC). The decrease in mortality risk due to reclassification in both groups was not confirmed statistically. Future studies should focus on statistical evidence when referring to the Will Rogers phenomenon, instead of inferring from raw comparisons.

## 1. Introduction

Liver cancer remains a global health concern because of its increasing burden worldwide [1]. It represents a grouping disorder that refers to several broad types of cancer, with cholangiocarcinoma (CC) being the second most common primary malignant tumor [1,2]. CC is known for its relatively low occurrence and poor prognosis, primarily driven by delayed detection [2].

CC is broadly classified as intrahepatic cholangiocarcinoma (ICC) or extrahepatic cholangiocarcinoma (ECC) based on anatomical location [3,4]. ECC includes distal CC (DCC) and perihilar CC (PCC, also known as Klatskin tumors) [5]. The latter is located at the bifurcation of the hepatic duct and has been associated with incorrect definitions [4,6,7]. Specifically, due to challenges in diagnosis and an imperfect coding system that distinguishes between PCC and DCC, the former is often miscoded as ICC rather than ECC, further affecting the estimates for both groups [4,7].

Current epidemiological findings on CC incidence and risk factors suggest geographical variation [8], with the highest and still increasing patterns observed in developing countries [7,9]. Furthermore, the existing literature highlights both the lack of comparative studies on ICC and ECC and the need for research in locations with the highest CC burden [4,10]. Acknowledging these limitations, we designed this study to analyze the surgical outcomes for all subtypes separately and compare ICC with ECC. In addition, we investigated the impact of subtype (re)classification in patients with CC and its effect on survival estimates, as determined by risk ratios (RR).

## 2. Materials and Methods

### 2.1. Study Design and Case Definitions

We conducted a retrospective study to evaluate the outcomes in the CC cohort following surgical treatment. We included all histologically confirmed cases that underwent liver resection at the National Research Oncology Centre, Astana, Kazakhstan (NROC). The eighth version of the AJCC Cancer Staging Manual was used to calculate the TNM stage grouping [11].

### 2.2. Data Source and Population

The NROC provided data for this study from January 2022 to July 2025. Each patient’s preoperative workup included a standard clinical examination, evaluation of serum laboratory tests, computed tomography, and/or magnetic resonance imaging. Surgery was only an option for patients whose tumors could be completely resected while leaving a functional liver remnant with adequate arterial inflow and hepatic venous outflow.

### 2.3. Outcome Assessment

Patients were contacted every three months in accordance with local protocols, and the NROC registry was updated with the relevant data. Survival status was defined using registry records, alive or dead from any cause (on 18 July 2025).

### 2.4. Variable Selection

Stepwise regression [12,13] and backward elimination [14,15] have been used in previous research to select variables when examining data from CC cohorts. However, existing evidence indicates that such variable processing may result in unstable inferences and induce systematic errors [16,17]. Therefore, we did not pre-filter the variables or eliminate weak effects. Instead, we relied on expert knowledge, including clinically important variables [18].

The registry made the following variables available: age (continuous scale), type of CC (e.g., ICC, DCC, PCC), sex (male, female), surgical margin (R0, R1), lymph node metastases (N0, N1, N2), and whether the percutaneous transhepatic biliary drainage (PTBD) was performed (yes, no). Categorical and continuous variables were presented as frequency (%) and median (interquartile range, IQR), respectively.

Of the 39 participants, 20 died. Given this number of events, we could only use four variables in the regression analysis to ensure an adequate events-per-variable ratio (EPV) [19]. Relying on the information criterion (e.g., AIC, BIC), neither age nor PTBD improved the model predictions. Therefore, we proceeded with the type of CC, sex, lymph node metastases, and surgical margin, which resulted in an acceptable EPV [19].

### 2.5. Statistical Analysis

Logistic regression is a conventional method for modeling cross-sectional data. However, its measure (i.e., Odds Ratio, OR) is prone to overstate the association between the predictor and outcome, particularly when an outcome is prevalent, requiring modified models [20,21]. The (common) outcome in our study was evident, namely, 51.28%. Furthermore, our sample size was small (*n* = 39). *p*-values are particularly sensitive to small deviations in such data, leading to ambiguous conclusions [22]. Therefore, we employed Poisson regression models with modified errors to calculate the risk ratios (RR) for all-cause mortality [23]. Unlike the OR, the RR can explicitly relate exposure to the outcome, further easing the interpretation. We also report marginal effects and its multiple comparisons incorporated Bonferroni corrections. *p*-values were two-sided, with a significance level of *p* < 0.05. Coefficient estimates and variance inflation factors (VIF) were used to check for multicollinearity, and the model fit was evaluated using Pearson’s goodness-of-fit test statistics. Data analysis was performed using STATA (V. 18).

### 2.6. Reclassification Analysis

Prior research has emphasized the consequences of incorrectly classifying Klatskin tumors as ICC in epidemiological studies. Here, we aimed to understand the effect of subtype (re)classification and investigate whether it affects the survival estimates. We began by modeling Klatskin tumors as ECC, contrasting the estimates to ICC (Model 1). We then reclassified them as ICC and compared ICC (and PCC) with DCC alone (Model 2) [6]. Finally, we compared the estimates obtained from the two models. We used the Bland–Altman approach to calculate Standard Errors (SE) for estimates [24], and the difference between the two model estimates was calculated as follows: (β1 − β2)/[sqrt(SE1)^2^ + (SE2)^2^]. To compare the differences in marginal effects between the reclassification models, we used bootstrapping (500 replications) to obtain the SE, CI, and *p*-values for the contrasts.

## 3. Results

A total of 39 patients were included in this study (Table 1). The median age at the time of surgery was 63 years (IQR: 54–69 years). Among them, 23 (58.97%) were male and 16 (41.03%) were female patients. The median follow-up time was 11.3 months (IQR: 5.7–21.8), and the median time to outcome was 188.7 days (IQR: 50.2–331.8). All patients underwent lymphadenectomy. The distributions of the CC subtypes were comparable. Additional clinicopathological characteristics are presented in Table 1 and in Supplemental Tables S1 and S2.

**Figure 1:**
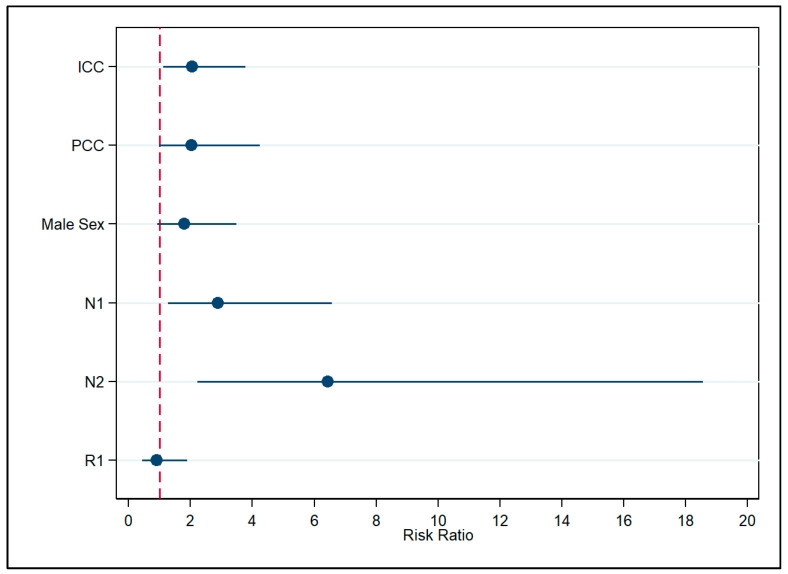
Association of covariates with death, each subtype being analyzed as they are, without grouping as intrahepatic or extrahepatic cholangiocarcinoma. Note: ICC—Intrahepatic Cholangiocarcinoma; PCC—Perihilar Cholangiocarcinoma; N1—Regional Lymph Node Involvement; N2—Extensive Regional Lymph Node Metastasis Involving Multiple Regional Nodes; R1—Positive Surgical Margin; VIF: 1.63; Pearson’s goodness-of-fit test statistics: χ^2^ = 17.44, df = 32, *p* = 0.98.

Analyzing each CC subtype separately, our regression analysis revealed an increased risk of death for patients with ICC (RR  =  2.05, 95% CI 1.11–3.78), PCC (RR  =  2.03, 95% CI 0.97–4.24), male sex (RR  =  1.80, 95% CI 0.93–3.49), LNM N1 (RR  =  2.88, 95% CI 1.27–6.57), N2 (RR  =  6.44, 95% CI 2.23–18.56); however, positive surgical margin indicated decreased risks (RR  =  0.91; 95% CI 0.43–1.90), when compared to DCC, female sex, absence of lymph node involvement, and negative surgical margin (Figure 1, Supplemental Table S3). Estimated marginal effects were 0.27 (95% CI 0.12–0.43), 0.56 (95% CI 0.29–0.83) and 0.55 (95% CI 0.22–0.89) for DCC, ICC and PCC, respectively. Further pairwise comparison did not reveal a notable difference between the subtypes (Supplemental Table S4).

**Figure 2:**
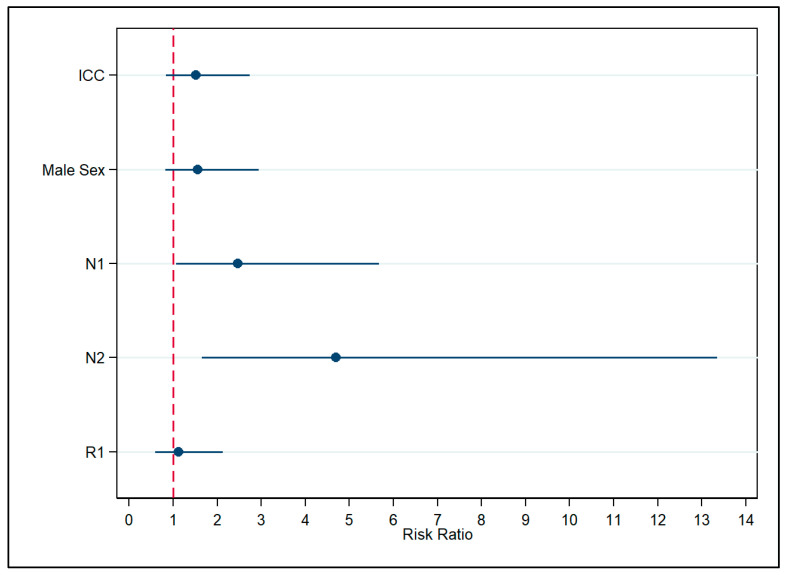
Association between covariates and death was assessed, with Klatskin tumors classified as extrahepatic cholangiocarcinoma. Note: ICC—Intrahepatic Cholangiocarcinoma; N1—Regional Lymph Node Involvement; N2—Extensive Regional Lymph Node Metastasis Involving Multiple Regional Nodes; R1—Positive Surgical Margin; VIF: 1.52; Pearson’s goodness-of-fit test statistics: χ^2^ = 19.16, df = 33, *p* = 0.97.

When analyzing Klatskin tumors as ECC, the estimates for most covariates showed a slight decrease and were as follows: ICC (RR  =  1.52, 95% CI 0.84–2.73), male sex (RR  =  1.55, 95% CI 0.82–2.94), LNM N1 (RR  =  2.46, 95% CI 1.07–5.67), N2 (RR  =  4.69, 95% CI 1.65–13.34), R0 (RR  =  1.12; 95% CI 0.58–2.13), when compared to ECC, female sex, absence of lymph node involvement, and negative surgical margin (Figure 2, Supplemental Table S5).

**Figure 3:**
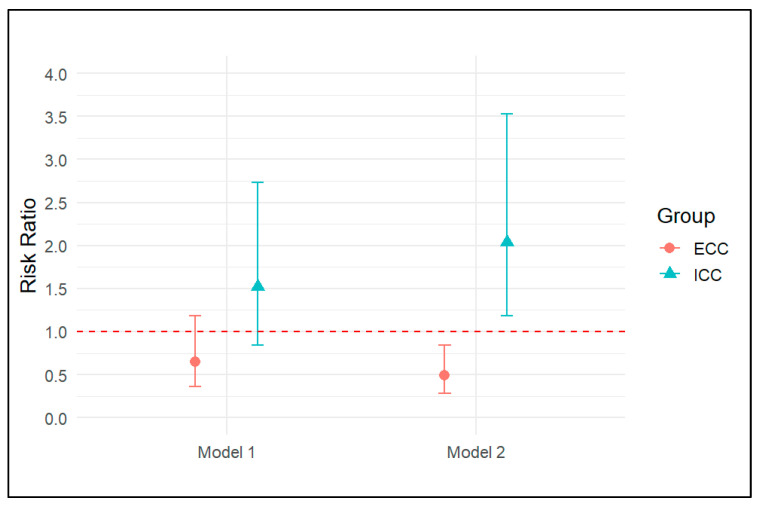
Estimated Risk Ratios of outcome from the reclassification analysis. Note: ICC—Intrahepatic Cholangiocarcinoma; ECC—Extrahepatic Cholangiocarcinoma; Model 1—Klatskin tumors modeled as ECC; Model 2—Klatskin tumors modeled as ICC.

**Figure 4:**
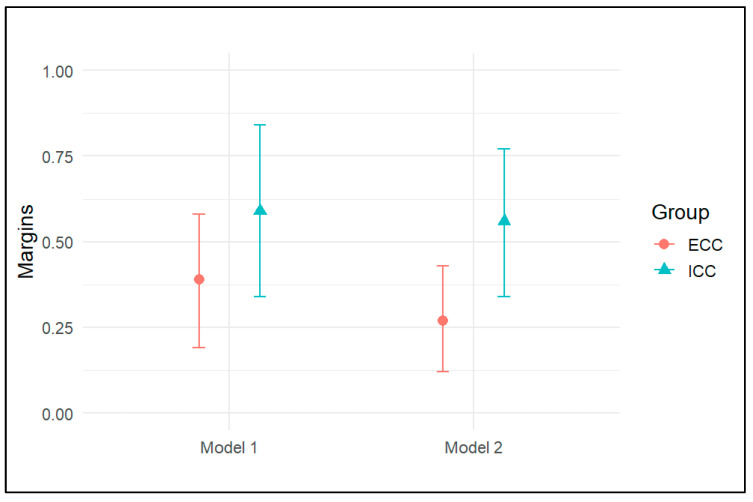
Estimated marginal effects of outcome from the reclassification analysis.

With reference to the reclassification analysis, Model 1 revealed an RR of 1.52 for ICC (95% CI: 0.84–2.73, *p* = 0.17). In Model 2, the estimated risk of death increased, with an RR of 2.04 (95% CI: 1.53–3.53, *p* = 0.01) (Figure 3). However, the post hoc analysis revealed insufficient evidence to suggest a difference between these estimates (z-value = 0.39, *p* = 0.69). A similar pattern was observed for ECC (z-value = 0.72, *p* = 0.47). Similarly, the adjusted marginal effects showed a reduction in the probability of death for both ICC and ECC (Figure 4). Specifically, for ICC, the probability decreased from 0.59 (95% CI 0.34–0.84) in Model 1 to 0.56 (95% CI 0.34–0.77) in Model 2. For ECC, the probability decreased from 0.39 (95% CI 0.19–0.58) in Model 1 to 0.27 (95% CI, 0.12–0.43) in Model 2. However, the difference was not meaningful and was 0.03 (95% CI −0.18–0.25, *p* = 0.75) and 0.11 (95% CI −0.03–0.27, *p* = 0.12) for ICC and ECC, respectively.

## 4. Discussion

This study evaluated the surgical outcomes of patients with CC, focusing on the prognostic impact of certain tumor-specific factors on survival. We revealed an elevated risk of death for ICC and PCC compared to DCC. A further comparison of ICC and ECC (combining PCC and DCC) did not reveal clear evidence of elevated risk. Finally, the reclassification of Klatskin tumors as ICC resulted in a decrease in the probability of death in both ECC and ICC, although the difference showed insufficient evidence for improved survival.

The existing literature suggests that CC subtypes are distributed unevenly, with PCC accounting for approximately half of all CC cases [25]. Furthermore, resectability rates vary among the subtypes, with different clinical prognoses reported [25,26]. Our study included only resected cases, and the distribution of the CC subtypes was comparable. This discrepancy may reflect the current trend toward increased resection rates, with 90% of patients with DCC and 80% of those with ICC and PCC undergoing curative liver resection [27,28]. We revealed better survival for DCC than for ICC and PCC, which aligns with existing evidence [26,29,30]. Moreover, a recent meta-analysis suggested that patients with DCC had paradoxically better survival despite being associated with the highest rate of LNM [29]. This also holds true for our study, as we observed improved survival for DCC, adjusting for sex, LNM, and surgical margin. Unlike multivariable analysis, the difference in survival was unclear in bivariate analysis. Notably, these observations are counterintuitive since lymphatic spread is normally associated with poor clinical prognosis [29]. Therefore, our findings should be interpreted with caution, considering the limitations of the study.

Surgical resection remains the mainstay treatment for CC. Negative (R0) resection is frequently the goal of curative-intent surgery [29]. Interestingly, in our study, surgical margins were not associated with discernible differences in survival. Its prognostic value for both recurrence-free survival and overall survival was also questioned in a meta-analysis of 2132 patients [18]. Nevertheless, the benefits of margin width have been consistently discussed in the literature and are likely to be multifactorial [31,32]. Therefore, our intention to interpret the effect of margin status while ignoring other clinically important factors (e.g., vascular invasion, tumor size, adjuvant treatment, and coexisting diseases) is somewhat questionable. For this reason, our findings are suggestive and require further evaluation.

A comparison of ICC and ECC (DCC and PCC combined) showed weak evidence of elevated relative risks in patients with ICC. The existing findings on this matter remain largely inconsistent. Ercolani et al. showed no difference in survival between ICC and ECC (combining PCC and DCC) [33]. However, a large longitudinal study revealed higher mortality risks in patients with ECC than in those with ICC [34]. Conversely, Mukkamalla et al. found that patients with ECC had a longer survival rate than those with ICC [35]. Finally, Guglielmi et al. found that ICC had better survival than ECC (PCC alone) [36]. The latter is consistent with another study claiming that patients with metastatic PCC “clearly do not benefit from resection” [37].While these subtypes may share common pathologies, inconsistent findings across studies may be attributed to variations in risk factors and tumor behavior. These factors may also confound comparisons between ICC and ECC, making it challenging to obtain reliable inferences regarding their clinical prognoses.

The cancer literature provides examples of how stage migration can lead to erroneous findings [38,39,40]. A paradox used to characterize this is referred to as the Will Rogers phenomenon (WRP). Although the WRP is often discussed in relation to stage migration or diagnostic drift, issues arising from reclassification (of CC subtypes) have rarely been the subject of interest.

We observed survival improvements in both groups when reclassifying PCC (from ECC) as ICC. At first glance, it may appear that there is a difference between the reclassified models. However, post estimations revealed that the difference was not close to being “statistically significant.” Similarly, a recent study analyzing the impact of misclassification (WRP) on epidemiological estimates by reclassifying PCC found an increase in ICC and a decrease in ECC, measured by the average annual percent change [6]. Another study examined the effects of WRP on survival function after switching from the AJCC 7th to the AJCC 8th edition for breast cancer staging [40]. Using the above approach, we found no evidence of meaningful changes in these studies.

In the original study that introduced the WRP to the medical field [38], Feinstein et al. observed improved six-month survival rates following stage migration when comparing two patient cohorts. Specifically, the (comparative) survival probabilities for stages I, II and III were 76% vs. 92%, 68% vs. 72%, and 36% vs. 42%, respectively. However, our statistical comparison of these differences yielded *p*-values of 0.07, 0.76, and 0.12 for these stages, respectively. On the one hand, by itself, statistical significance is insufficient for determining scientific or practical importance. However, studies frequently rely on significance testing when interpreting the findings resulting from reclassification/stage migration [6,38,40]. Hence, a quantitative measure might be required to contrast such changes. In light of our handful observations, we wonder if the comparison of estimates from two (reclassified) states mostly contradicts the WRP. Further research is required to address this concern.

### Limitations

Our study had several limitations. We were limited by the sample size and clinical information to propose reliable inferences. In addition, we may have model specification issues, implying that other clinically important variables were not considered in the analysis. Specifically, we did not have information on whether pancreaticoduodenectomy was performed, the presence of comorbid diabetes mellitus, hypertensive diseases, other metabolic syndrome components, and presurgical laboratory data that may potentially affect the surgical outcomes. Therefore, our findings are by no means conclusive. More data are required to address our concerns. Furthermore, the literature highlights issues related to the correct diagnosis of PCC [4,6]. Both ICC and ECC involving the hilar hepatic duct confluence may affect the accuracy of PCC estimates. There is also widespread confusion over terminology (e.g., “hilar,” “perihilar,” and “Klatskin”) and coding practices [6]. To date, no version of the ICD coding system has perfectly distinguished between PCC and DCC [7]. Prior research has emphasized that the majority of cancer registries label unspecified tumor sites as ICC [7]. Taken together, our CC subtypes might not be free of misclassifications, which may potentially affect all estimations. Therefore, our findings remain exploratory and should be interpreted with particular caution.

## 5. Conclusions

We found a slightly better survival prognosis for DCC than for ICC and PCC. There were no differences in postsurgical mortality between ECC and ICC in both bivariate and multiple regression analyses. The distinct decrease in mortality risks due to reclassification for both ICC and ECC did not find subsequent statistical confirmation in the present study. Future studies should focus on statistical evidence when referring to the Will Rogers phenomenon rather than drawing conclusions based on a qualitative perspective.

## Figures and Tables

**Table 1:** Baseline patient characteristics.

Covariate	Total (*n* = 39, 100.0%)	ICC (*n* = 13, 33.3%)	DCC (*n* = 14, 35.9%)	PCC (*n* = 12, 30.8%)	*p*-Value
**Age**	63 (IQR: 54–69)	62 (IQR: 59–66)	60 (IQR: 51–70)	61 (IQR: 51–65)	0.84
**Sex**					1.0
Female	16 (41.1%)	5 (38.6%)	6 (42.6%)	5 (41.7%)	
Male	23 (58.9%)	8 (61.4%)	8 (57.4%)	7 (58.3%)	
**LNM**					0.21
N0	17 (43.6%)	8 (61.5%)	3 (21.4%)	6 (50.0%)	
N1	19 (48.7%)	5 (38.5%)	9 (64.3%)	5 (41.7%)	
N2	3 (7.7%)	0 (0.00%)	2 (14.3%)	1 (8.3%)	
**Surgical margin**					0.04
R0	24 (61.4%)	11 (84.6%)	9 (64.3%)	4 (33.3%)	
R1	15 (38.6%)	2 (15.4%)	5 (35.7%)	8 (66.7%)	
**PTBD**					0.01
No	21 (53.8%)	12 (92.3%)	5 (35.7%)	4 (33.3%)	
Yes	18 (46.2%)	1 (7.7%)	9 (64.3%)	8 (66.7%)	
**Survival state**					0.78
Alive	19 (48.7%)	6 (46.2%)	8 (57.1%)	5 (41.7%)	
Dead	20 (51.3%)	7 (53.8%)	6 (42.9%)	7 (58.3%)	

Note: PTBD—Percutaneous Transhepatic Biliary Drainage; LNM: Lymph Node Metastases; IQR—Interquartile Range; ICC—Intrahepatic Cholangiocarcinoma; DCC—Distal Cholangiocarcinoma; PCC—Perihilar Cholangiocarcinoma.

## Data Availability

Due to its non-public nature, access to the dataset is limited and obtaining access requires contacting the National Research Oncology Centre.

## Supplementary Materials

The following supporting information can be downloaded at https://www.mdpi.com/article/10.3390/cancers17193263/s1, Table S1. Distribution of TNM classifications among clinical subgroups; Table S2. Distribution of stage classifications among clinical subgroup; Table S3. Association between covariates and death was analyzed separately for each cholangiocarcinoma subtype, without combining them into intrahepatic or extrahepatic groups; Table S4. Pairwise comparison of each cholangiocarcinoma subtype, without combining them into intrahepatic or extrahepatic groups; Table S5. Association between covariates and death was assessed, with Klatskin tumors classified as extrahepatic cholangiocarcinoma.

## Abbreviations

The following abbreviations are used in this manuscript:

AIC	Akaike Information Criterion
AJCC	American Joint Committee on Cancer
BIC	Bayesian Information Criterion
CC	Cholangiocarcinoma
CI	Confidence Interval
DCC	Distal Cholangiocarcinoma
ECC	Extrahepatic Cholangiocarcinoma
EPV	Event-per-variable
ICC	Intrahepatic Cholangiocarcinoma
IQR	Interquartile Range
LNM	Lymph Node Metastasis
NROC	National Research Oncology Center
PCC	Perihilar Cholangiocarcinoma
PTBD	Percutaneous Transhepatic Biliary Drainage
RR	Risk Ratio
SE	Standard Error
TNM	Tumor Nodes Metastases
VIF	Variance Inflation Factor
WRP	Will Rogers Phenomenon
