# Supplementary material for: Understanding the Will Rogers Phenomenon in Cholangiocarcinoma Research and Beyond
Source: Cancers (Basel). 2025 Oct 8;17(19):3263. doi: 10.3390/cancers17193263 (PMC12523421; doi:10.3390/cancers17193263)
Supplement: Supplementary file 1 [file cancers-17-03263-s001.zip › cancers-3864717-supplementary.pdf]

# Supplementary Materials: Understanding the Will Rogers Phenomenon in Cholangiocarcinoma Research and Beyond

**Ruslan Akhmedullin, Zhandos Burkitbayev, Tair Koishibayev, Zhanat Spatayev, Abylaikhan Sharmenov, Oxana Shatkovskaya, Dinara Zharlyganova, Almira Manatova, Zhuldyz Kuanysh, Sanzhar Shalekenov and Abduzhappar Gaipov**

**Table S1.** Distribution of TNM classifications among clinical subgroups.

| TNM | Total | ICC | DCC | PCC |
|---|---|---|---|---|
| pT2N0M0 | 1 (2.56%) | 1 (7.69%) | 0 (0.00%) | 0 (0.00%) |
| pT2N1M0 | 1 (2.56%) | 1 (7.69%) | 0 (0.00%) | 0 (0.00%) |
| T2N0M0 | 1 (2.56%) | 0 (0.00%) | 1 (7.14%) | 0 (0.00%) |
| T3N0M0 | 1 (2.56%) | 0 (0.00%) | 1 (7.14%) | 0 (0.00%) |
| T3N1M0 | 1 (2.56%) | 0 (0.00%) | 1 (7.14%) | 0 (0.00%) |
| T3N2M0 | 1 (2.56%) | 0 (0.00%) | 1 (7.14%) | 0 (0.00%) |
| pT2N0M0 | 1 (2.56%) | 1 (7.69%) | 0 (0.00%) | 0 (0.00%) |
| pT3N1M0 | 1 (2.56%) | 1 (7.69%) | 0 (0.00%) | 0 (0.00%) |
| pT2aN1M0 | 1 (2.56%) | 1 (7.69%) | 0 (0.00%) | 0 (0.00%) |
| T1N1M0 | 1 (2.56%) | 0 (0.00%) | 1 (7.14%) | 0 (0.00%) |
| T1bN0M0 | 1 (2.56%) | 1 (7.69%) | 0 (0.00%) | 0 (0.00%) |
| T2N0M0 | 1 (2.56%) | 1 (7.69%) | 0 (0.00%) | 0 (0.00%) |
| T2bN1M0 | 1 (2.56%) | 0 (0.00%) | 0 (0.00%) | 1 (8.33%) |
| T3N1M0 | 2 (5.13%) | 0 (0.00%) | 1 (7.14%) | 1 (8.33%) |
| T3N1MO | 1 (2.56%) | 0 (0.00%) | 1 (7.14%) | 0 (0.00%) |
| T3N2M1 | 1 (2.56%) | 0 (0.00%) | 0 (0.00%) | 1 (8.33%) |
| pT2N0M0 | 1 (2.56%) | 1 (7.69%) | 0 (0.00%) | 0 (0.00%) |
| pT2N1M0 | 1 (2.56%) | 1 (7.69%) | 0 (0.00%) | 0 (0.00%) |
| pT2bN0M0 | 1 (2.56%) | 0 (0.00%) | 0 (0.00%) | 1 (8.33%) |
| T1N0M0 | 1 (2.56%) | 0 (0.00%) | 1 (7.14%) | 0 (0.00%) |
| T3N0M0 | 1 (2.56%) | 1 (7.69%) | 0 (0.00%) | 0 (0.00%) |
| T3N1M0 | 3 (7.69%) | 0 (0.00%) | 3 (21.43%) | 0 (0.00%) |
| T3N1M0 | 1 (2.56%) | 0 (0.00%) | 1 (7.14%) | 0 (0.00%) |
| T3N1M1 | 1 (2.56%) | 0 (0.00%) | 1 (7.14%) | 0 (0.00%) |
| T3N2M0 | 1 (2.56%) | 0 (0.00%) | 1 (7.14%) | 0 (0.00%) |
| T4N0M0 | 1 (2.56%) | 0 (0.00%) | 0 (0.00%) | 1 (8.33%) |
| T4N1M0 | 1 (2.56%) | 1 (7.69%) | 0 (0.00%) | 0 (0.00%) |
| pT1N0M0 | 1 (2.56%) | 1 (7.69%) | 0 (0.00%) | 0 (0.00%) |
| pT2N0M0 | 1 (2.56%) | 1 (7.69%) | 0 (0.00%) | 0 (0.00%) |
| pT2bN0M0 | 3 (7.69%) | 0 (0.00%) | 0 (0.00%) | 3 (25.00%) |
| pT2bN1M0 | 1 (2.56%) | 0 (0.00%) | 0 (0.00%) | 1 (8.33%) |
| pT2bN2M0 | 1 (2.56%) | 0 (0.00%) | 0 (0.00%) | 1 (8.33%) |
| pT3N1M0 | 1 (2.56%) | 0 (0.00%) | 0 (0.00%) | 1 (8.33%) |
| pT4N1M0 | 1 (2.56%) | 0 (0.00%) | 0 (0.00%) | 1 (8.33%) |

**Table S2.** Distribution of stage classifications among clinical subgroups.

| Stage | Total | ICC | DCC | PCC |
|---|---|---|---|---|
| IV | 1 (2.56%) | 0 (0.00%) | 1 (7.14%) | 0 (0.00%) |
| IVA | 2 (5.13%) | 0 (0.00%) | 0 (0.00%) | 2 (16.67%) |

| | | | | |
|---|---|---|---|---|
| IVA. | 1 (2.56%) | 0 (0.00%) | 1 (7.14%) | 0 (0.00%) |
| Iva | 2 (5.13%) | 1 (7.69%) | 0 (0.00%) | 1 (8.33%) |
| St. Ivb | 1 (2.56%) | 0 (0.00%) | 0 (0.00%) | 1 (8.33%) |
| IIIc | 3 (7.69%) | 1 (7.69%) | 0 (0.00%) | 2 (16.67%) |
| IIIC | 1 (2.56%) | 0 (0.00%) | 0 (0.00%) | 1 (8.33%) |
| IIIA | 1 (2.56%) | 0 (0.00%) | 1 (7.14%) | 0 (0.00%) |
| IIIb | 1 (2.56%) | 1 (7.69%) | 0 (0.00%) | 0 (0.00%) |
| IIIB | 3 (7.69%) | 2 (15.38%) | 0 (0.00%) | 1 (8.33%) |
| IIIa | 1 (2.56%) | 1 (7.69%) | 0 (0.00%) | 0 (0.00%) |
| III | 1 (2.56%) | 0 (0.00%) | 0 (0.00%) | 1 (8.33%) |
| IIB | 2 (5.13%) | 0 (0.00%) | 2 (14.29%) | 0 (0.00%) |
| IIB | 3 (7.69%) | 0 (0.00%) | 3 (21.43%) | 0 (0.00%) |
| IIb | 1 (2.56%) | 0 (0.00%) | 1 (7.14%) | 0 (0.00%) |
| IIB | 2 (5.13%) | 0 (0.00%) | 2 (14.29%) | 0 (0.00%) |
| IIA | 1 (2.56%) | 0 (0.00%) | 1 (7.14%) | 0 (0.00%) |
| Iia | 1 (2.56%) | 0 (0.00%) | 1 (7.14%) | 0 (0.00%) |
| II | 8 (20.51%) | 5 (38.46%) | 0 (0.00%) | 3 (25.00%) |
| ST II | 1 (2.56%) | 1 (7.69%) | 0 (0.00%) | 0 (0.00%) |
| I | 2 (5.13%) | 1 (7.69%) | 1 (7.14%) | 0 (0.00%) |
| Iib | 1 (2.56%) | 0 (0.00%) | 1 (7.14%) | 0 (0.00%) |

**Table S3.** Association between covariates and death was analyzed separately for each cholangiocarcinoma subtype, without combining them into intrahepatic or extrahepatic groups.

| Covariate | Model 1. Crude RR and 95% CI | *p*-Value | Model 1. Adjusted RR and 95% CI | *p*-Value |
|---|---|---|---|---|
| **CC Subtype** | | | | |
| DCC | 1.0 | | 1.0 | |
| ICC | 1.25 (0.65–2.78) | 0.57 | 2.05 (1.11–3.78) | 0.02 |
| PCC | 1.36 (0.62–2.97) | 0.44 | 2.03 (0.97–4.24) | 0.08 |
| **Sex** | | | | |
| Female | 1.0 | | 1.0 | |
| Male | 1.29 (0.66–2.52) | 0.45 | 1.80 (0.93–3.49) | 0.08 |
| **LNM** | | | | |
| N0 | 1.0 | | 1.0 | |
| N1 | 2.15 (0.94–4.89) | 0.06 | 2.88 (1.27–6.57) | 0.01 |
| N2 | 3.40 (1.61–7.16) | 0.001 | 6.44 (2.23–18.56) | 0.001 |
| **Surgical margin** | | | | |
| R0 | 1.0 | | 1.0 | |
| R1 | 1.31 (0.71–2.40) | 0.38 | 0.91 (0.43–1.90) | 0.79 |

VIF: 1.63; Pearson goodness-of-fit test statistics: $\chi^2 = 17.44$, df = 32, $p = 0.98$.

**Table S4.** Pairwise comparison of each cholangiocarcinoma subtype, without combining them into intrahepatic or extrahepatic groups.

| Subtype | Contrast (SE) | Bonferroni | | Bonferroni 95% CI |
|---|---|---|---|---|
| | | z | *p*-Value | |
| ICC vs. DCC | 0.29 | 2.16 | 0.09 | −0.03–0.61 |
| PCC vs. DCC | 0.28 | 1.63 | 0.31 | −0.13–0.70 |
| PCC vs. ICC | −0.01 | −0.03 | 1.00 | −0.55–0.53 |

**Table S5.** Association between covariates and death was assessed, with Klatskin tumors classified as extrahepatic cholangiocarcinoma.

| Covariate | Model 1. Crude RR and 95% CI | *p*-Value | Model 1. Adjusted RR and 95% CI | *p*-Value |
|---|---|---|---|---|
| **CC Subtype** | | | | |
| ECC | 1.0 | | 1.0 | |
| ICC | 1.10 (0.56–2.04) | 0.82 | 1.52 (0.84–2.73) | 0.16 |
| **Sex** | | | | |
| Female | 1.0 | | 1.0 | |
| Male | 1.29 (0.66–2.52) | 0.45 | 1.55 (0.82–2.94) | 0.17 |
| **LNM** | | | | |
| N0 | 1.0 | | 1.0 | |
| N1 | 2.15 (0.94–4.89) | 0.06 | 2.46 (1.07–5.67) | 0.03 |
| N2 | 3.40 (1.61–7.16) | 0.001 | 4.69 (1.65–13.34) | 0.004 |
| **Surgical margin** | | | | |
| R0 | 1.0 | | 1.0 | |
| R1 | 1.31 (0.71–2.40) | 0.38 | 1.12 (0.58–2.13) | 0.73 |

VIF: 1.52; Pearson goodness-of-fit test statistics: $\chi^2 = 19.16$, df = 33, $p = 0.97$.
